# Towards universal coverage—highlights from the 2nd Uganda Conference on Cancer and Palliative Care, 5–6 September 2019, Kampala, Uganda

**DOI:** 10.3332/ecancer.2019.976

**Published:** 2019-11-19

**Authors:** Julia Downing, Nixon Niyonzima, Sam Guma, Mwazi Batuli, Rose Kiwanuka, Innocent Atuhe, Zaitun Nalukwago, Mark Mwesiga, Warren Phipps, Henry Ddungu

**Affiliations:** 1 Makerere University and International Children’s Palliative Care Network, Kampala, Uganda; 2 Uganda Cancer Institute, Kampala, Uganda; 3 Kawempe Home Care, Kampala, Uganda; 4 Islamic University in Uganda (IUIU), Kampala, Uganda; 5 Palliative Care Association of Uganda, Kampala, Uganda; 6 Uganda Cancer Institute—Fred Hutch, Kampala, Uganda

**Keywords:** Cancer care, palliative care, Uganda, policy, universal health coverage, research, paediatrics, sustainable development goals

## Abstract

The 2nd Uganda Conference on Cancer and Palliative Care was held in September 2019 in Kampala, Uganda under the theme: Towards Universal Coverage. It was hosted by the Uganda Cancer Institute and the Palliative Care Association of Uganda (PCAU). The conference brought together 350 delegates from eight countries. Key themes from the conference included: universal health coverage (UHC), service provision and public health; resources for achieving UHC; capacity building; human rights and engagement on the implementation of the recommendations made by the Uganda Human Rights Commission; provision of cancer and palliative care to ‘hard to reach’ and ‘vulnerable’ groups; paediatrics; health promotion and prevention; policy and advocacy and digital technology. The conference also gave opportunity to celebrate the 20th Anniversary of the work of PCAU, with a celebration dinner attended by the Minister of Health. The past few years have seen significant developments in both cancer and palliative care in Uganda, and this was evident in the presentations, and the way that provision has changed and improved since the first cancer and palliative care conference in 2017. Emphasis on UHC, along with the support of government and other stakeholders, is important in the ongoing development of cancer and palliative care services in Uganda.

## Introduction

Universal health coverage (UHC) means that ‘*all people and communities can use the promotive, preventive, curative, rehabilitative and palliative health services they need, of sufficient quality to be effective, while also ensuring that the use of these services does not expose the user to financial hardship’* [1]. It is part of the Sustainable Development Goal (SDG) 3 ‘Good Health and Well-Being’ adopted by the United Nations Member States in 2015 to be achieved by 2030 [2]. The SDGs are inter-related and the achievement of one will impact on the achievement of others [2]. In the attempt to ‘Leave no-one behind’, UHC addresses two key elements: (1) ensuring people can access the health services that they need and (2) the economic consequences of individuals accessing health services [3]. This 2nd Uganda Conference on Cancer and Palliative Care, organised by the Uganda Cancer Institute (UCI) and the Palliative Care Association of Uganda (PCAU), covered all aspects of cancer and palliative care, with a focus on UHC and making sure that individuals across Uganda will be able to access the full spectrum of cancer and palliative care service provision without causing financial hardship.

The conference was also a celebration of 20 years of advocacy and co-ordination for palliative care in Uganda from PCAU and 25 years of palliative care service provision in Uganda. Palliative care is ‘*an approach that improves the quality of life of patients and their families facing the problem associated with life-threatening illness, through the prevention and relief of suffering by means of early identification and impeccable assessment and treatment of pain and other problems, physical, psychosocial and spiritual*’ [4]. At the heart of this joint UCI-PCAU conference was the commitment to improve access to cancer care and palliative care within Uganda through UHC, with a desire to share and learn from each other.

PCAU is the National Association for Palliative Care in Uganda established in 1999 and registered as a non-governmental organisation (NGO) in 2003 to support and promote the development of palliative care in Uganda. Currently, PCAU is composed of 21 member organisations and 1,056 individual members. PCAU works in partnership with the Ministry of Health, other line government ministries, agencies, departments, civil society and individuals to accelerate the integration of palliative care into the health care system in Uganda. PCAU’s vision is ‘Palliative Care for all in need in Uganda’ with a mission to accelerate the integration of palliative care in the Uganda healthcare system through capacity building, advocacy, research and resource mobilisation. With the emphasis on the SDGs and UHC, PCAU is focusing on working with the government and civil society to ensure that no one is left behind and everyone in Uganda has access to palliative care when needed.

The UCI was founded in 1967 through a joint venture with the American National Cancer Institute, the Makerere Department of Surgery and the British Empire Cancer Campaign. UCI’s functions include but are not limited to: (1) developing policy on the prevention, diagnosis and treatment for cancers and on the care for patients with cancer and cancer-related diseases; (2) undertaking and coordinating the prevention and treatment of cancers in Uganda; (3) providing comprehensive medical care services to patients affected with cancer and cancer-related diseases; (4) providing palliative care and rehabilitation services to patients with cancer; (5) overseeing the management of cancer and cancer-related services in public and private health centres and (6) conducting or coordinating cancer-related research activities in Uganda and outside Uganda. UCI registers an average of 4,500 new patients annually and is a centre of excellence in research and clinical care.

## Conference background

Uganda has a high disease burden of both communicable diseases and non-communicable diseases (NCDs) [5] with high morbidity and mortality [6]. In 2018, there were 32,617 new cancer cases in the country, with 21,829 cancer deaths [7]. Alongside this, 80% of individuals with cancer in Uganda either report or are diagnosed late, making cure an option that is still far from reality, and the need for palliative care an important priority [8]. Uganda is seen as one of the leading countries in sub-Saharan Africa for the provision of palliative care [9–11]. The 2030 Agenda for Sustainable Development [2] and the SDGs, in particular that of Goal 3: Good health and well-being [12], including UHC, are important opportunities for enhancing cancer and palliative care service provision.

These themes were highlighted in the opening plenary of the conference where Dr E. Luyirika (African Palliative Care Association) and Dr Yonas Tegegn Woldemariam’s representative (World Health Organization) noted the importance of UHC and leaving no one behind, highlighting the status of cancer and palliative care service provision within Uganda, encouraging members to participate in this. Key initiatives along with UHC were discussed, such as the WHA resolutions on cancer [13] and palliative care [14], the Astana Declaration on Primary care [15] and the Lancet Commission report on alleviating the access abyss to palliative care and pain relief [16]. Together they highlighted some of the areas where we still need to focus our energies, along with those where we are moving forward, for example, the limited access to radiotherapy in many countries across Africa, including Uganda [17–19] and the developments that are happening with regard to expanding radiotherapy access in Uganda with the UCI bringing in a linear accelerator to work alongside the existing cobalt machine. Dr E. Luyirika also challenged the Ministry of Health with regard to financing for both cancer and palliative care services, and the Minister responded by outlining plans for the new health insurance scheme being implemented in Uganda.

Political will for UHC and the expansion of cancer and palliative care throughout the country were confirmed by the Minister of Health, the Hon Dr Ruth Jane Aceng and the Prime Minister of the Republic of Uganda, the Rt Hon Dr Ruhakana Rugunda who officially opened the conference. The Minister of Health discussed how Uganda is undergoing a demographic transition and that there is a lot of interplay between communicable and NCDs. The Health Minister discussed the new National Health Insurance Scheme, reassuring participants that cancer and palliative care service provision has been included within the insurance scheme. Dr Aceng further stated that palliative care is now allocated a full division in the MoH and they have included palliative care consultant positions in the three major hospitals in Kampala (Mulago, Kawempe and Kiruddu). The Ministry of Health also intends to open up cancer centres in all regions in the country, initially targeting regions where there are universities and that UCI now has a mandate to procure cancer medicines. The Ministry is also committed to ensuring that there are adequately trained personnel, with training on both cancer and palliative care taking place.

When opening the conference, the Prime Minister, the Rt Hon Dr Ruhakana Rugunda discussed some of the strategies that are being utilised to strengthen cancer and palliative care service provision, including: (1) training specialists in cancer care for the country through UCI; (2) improving access to cancer medicines and supplies; (3) improving access to radiotherapy services; (4) improving access to palliative care services; (5) expanding cancer prevention and screening programmes; (6) taking cancer services closer to the people through the establishment of regional cancer centres and (7) protecting patients from catastrophic health expenditure, currently >40% is out of pocket expenditure by households and so Ugandans are overburdened by the cost of health care and so the new health insurance scheme should help with this. However, he acknowledged that there are challenges to the provision of cancer and palliative care through UHC including: (1) finances and resources; (2) an overreliance on direct payments at the time people need care, e.g., fees, medicines, etc. and (3) the inefficient use of resources, with 20%–40% of health resources being wasted worldwide and reducing waste would improve the ability of health systems to provide quality services and improve health.

Ultimately, he said that the aim in Uganda is to leave no one behind and then declared the conference officially open.

## Conference summary

The conference, held at the Kampala Serena Hotel, brought together 350 delegates from eight countries, with delegates from: Austria, Ethiopia, Ireland, Malawi, Uganda, United Kingdom, the United States and Zimbabwe. It brought together clinicians, academics, human rights advocates, lawyers, clergy, researchers, social workers, policy makers, Ministry of Health officials and donors, representing over 100 organisations, to share lessons and adopt best practice for cancer and palliative care and followed on from the previous successful conference held in 2017 [11]. Twenty organisations, both national, regional and international had exhibition stands, the media were present and interpreters translated the conference into sign language.

The conference was organised into six tracks: (1) capacity building; (2) service provision; (3) paediatrics; (4) health promotion and prevention; (5) policy and advocacy and (6) research and innovation. The scientific programme included a variety of plenary sessions (15 papers plus panel discussions), 47 oral breakout presentations, 3 workshops and 25 poster presentations over the 2 days. Presentations were given across the continuum of care, across the age span, for vulnerable and hard to reach groups, and while the main focus was on cancer care, the provision of palliative care for other conditions was also addressed.

During the dinner, which celebrated the 20th anniversary of PCAU, Rose Kiwanuka, Country Director for PCAU shared the history of PCAU, documenting its evolution from being formed and housed at Hospice Africa Uganda, employing the first staff, moving to being hosted by the African Palliative Care Association (APCA), to renting their own offices on Makindye Hill, through to moving to their own facility in Kitende on the Entebbe Road. Dr V. Walusansa, Deputy Executive Director UCI, congratulated PCAU on their 20 years and shared the latest achievements and plans of UCI.

The Hon Dr J. R. Aceng, the Minister of Health for the Republic of Uganda gave a short speech and encouraged everyone to enjoy the dinner and dancing. She also presented Awards from PCAU presented to individuals and organisations in recognition of their contribution to palliative care in Uganda. These awards went to: Dr J. Amandua for his commitment to palliative care while serving as a Commissioner in the Uganda Ministry of Health; and Mr R. Segawa for his work in founding Rays of Hope Hospice Jinja. Awards were also presented to the Centre for Hospice Care (USA) for continuous partnership with PCAU, Dr A. Ocero for his contribution to the spread of palliative care services in Uganda, Mr T. Duku for his contribution to the founding of PCAU and Hospice Tororo for their great contribution to the spread of palliative care services in the Eastern Region of Uganda. Awards were also given to individuals from UCI for their distinguished work in the care of cancer patients in Uganda. The Celebration dinner was rounded off by music from Ziwuuna Band accompanied by dancing.

## Key conference themes

The key themes identified from the conference were as follows.

1. UHC, service provision and public health.
2. Resources for achieving UHC.
3. Capacity building.
4. Human rights and engagement on the implementation of the recommendations made by the Uganda Human Rights Commission.
5. Provision of cancer and palliative care to ‘hard to reach’ and ‘vulnerable’ groups.
6. Paediatrics.
7. Health promotion and prevention.
8. Policy and advocacy.
9. Digital technology.

Alongside these, there were many cross-cutting issues identified across the conference such as the need for good communication, research and data, integration of services, compassion and collaboration.

### UHC, service provision and public health

Throughout the conference, the importance of the SDGs and UHC was stressed, and ensuring the integration of cancer and palliative care services within UHC. The importance of the WHA resolutions on cancer [13] and on palliative care [14] was also acknowledged, along with the influence of the Lancet Commission report on palliative care [16]. The scene was set for the conference through hearing from three direct stakeholders about their experiences in receiving cancer and palliative care. It was inspirational to hear their stories, but it was also acknowledged that they were all examples of individuals who had been able to access cancer and palliative care services and that there are many in Uganda who cannot, who are being ‘left behind’.

Professor F. Ssengooba, from the School of Public Health at Makerere University, discussed UHC from a Public Health Perspective identifying key priorities including: (1) the need for main policy changes in order to implement the UHC roadmap; (2) the fast-growing population in Uganda not matched by investments; (3) persistence of a high disease burden in Uganda; (4) deficits in access to and quality of services; (5) inadequate financing for health programmes and (6) capacity gaps in the community and decentralisation of the service delivery system.

There then followed several breakaway sessions addressing this issue. Within cancer care, various papers were presented by the UCI, including the palliative care needs and outcomes of patients with hepatocellular carcinoma, factors affecting chemotherapy adherence among breast cancer patients, multi-drug resistant neutropenia in individuals with haematological malignancies, attendant and caregiver temperature surveillance for cancer patients and predictors of Kaposi Sarcoma immune reconstitution syndrome. Within palliative care, topics discussed included: the palliative care needs and outcomes of individuals with hepatocellular carcinoma, assessment of the readiness and availability of palliative care services in hospitals in Kampala, experience of the Pain-Free Hospital Initiative (PFHI) in a national referral psychiatric hospital, reducing psychosocial issues through income generation, the impact of palliative care in advanced HIV disease and the use of volunteers in palliative care service provision. Professor A. Merriman challenged everyone in her presentation entitled ‘Audacity to Love’ as to our motivation behind the work that we do and the need to show love and compassion to all [20].

Pain management is an important component of both cancer and palliative care and a workshop was held at the conference on sustainable pain management in hospitals in Uganda. Since 1993, Uganda has seen improvement in access to pain medicines for palliative care patients by ensuring certain medications are on the Essential Medicines lists. Uganda has also developed a model for the local production of low-cost oral liquid morphine that has been instrumental in improving access to pain medicine among palliative care patients [21]. There is a public–private partnership (PPP) for the importation, manufacture and distribution of morphine in the country. Government funds the manufacture of the medicine at Hospice Africa Uganda (HAU) and this is distributed to public health facilities by the National Medical Stores (NMS), and to private facilities by the Joint Medical Stores (JMS). The 2004 task shifting instrument that allowed palliative care trained nurses to prescribe certain analgesics including oral liquid morphine also contributed to the availability of morphine which, according to the Minister’s speech at the conference, is now available to 234 health facilities in the country. To add to the efforts of improving the quality and access to pain management, PCAU in partnership with the Ministry of Health has implemented the PFHI in 14 hospitals in Uganda since 2015. The PFHI is a 1-year hospital-wide quality improvement initiative to integrate pain treatment into routine hospital care. Within the workshop, the experiences, lessons learnt, scale-up and sustainability of the initiative were discussed with the development of an action plan for the scalability and sustainability of the initiative in Uganda.

Throughout the presentations, the themes of ensuring that individuals can access care near to home were stressed, and how the provision of UHC can enable this to happen. An awareness of the political environment both within Uganda, the surrounding region and globally, was acknowledged as key in increasing access to and quality of service provision, and in advocating for ongoing support from the government, while recognising that already being provided.

### Resources for achieving UHC in Uganda

Following the opening of the conference, a panel discussion was held addressing the issue of what are the critical resources for achieving UHC for cancer and palliative care services in Uganda. The panel included: Dr C. Olaro, Director Health Services (Clinical and Community), Ministry of Health, Proscovia Ayebare, Dr E. Luyirika, Dr V. Walusansa, R. Kiwanuka and T. Twembi, Principal Human Rights Officer, Uganda Human Rights Commission. A wide variety of questions were posed to the panelists, including those on the government plans to achieve UHC, the resources in place and how the proposed National Health Insurance Scheme will help in ensuring access to cancer and palliative care services; what we can learn from other countries, how is the Ugandan Human Rights Commission taking steps to ensure its recommendations are being heeded?. While the panel discussion was shortened due to time constraints, it was informative and helpful in addressing the issue of resources.

On the second day of the conference, Professor F. Omaswa from the African Centre for Global Health and Social Transformation (ACHEST) addressed the issue of creating an appropriate skill mix for cancer and palliative care service delivery. He discussed the importance of capacity building and ensuring the appropriate skill mix, along with the fact that we all have a role to play in this. He noted three key issues: (1) it is important that we have strong organisation within ourselves, lets meet regularly, have a clear workplan, ensure that leadership is coming from us—we need to make UHC happen, ensuring no one is left behind; (2) we need to provide individuals within the health structures with the knowledge they need to remain healthy and (3) we need to make sure that our work is of the highest quality and is at the policy level. He reminded us that we have the skills and resources, so we need to go and do it!

Capacity building for cancer and palliative care is essential if we are to achieve UHC. This theme was continued by Dr S. Musene, Commissioner of Business, Technology and Vocational Education and Training (BTVET), from the Ministry of Education who shared as to how palliative care has been incorporated into nursing training institutions, in particular, at Mulago School of Nursing and Midwifery where the Advanced Diploma in Palliative Nursing, approved in 2018, is now being run, with the second cohort of nurses being trained. This is an important milestone in the delivery of palliative care education for nurses in the country as it is now embedded in the government system, thus impacting on sustainability.

### Capacity building

It is recognised that in order to extend cancer and palliative care services through UHC, capacity building is crucial. It is acknowledged in the WHA resolution on palliative care [14] that capacity building is needed at several levels including: (1) basic training and continuing education; (2) intermediate training and (3) specialist training. This is similar for cancer care; however, it also needs to be extended beyond health professionals to families, the media, community health workers, volunteers, etc. such that care can be provided across the continuum and individuals can access care from trained individuals. A range of options were discussed for building capacity at different levels, including the training of Village Health teams, the establishment of the advanced diploma in palliative care nursing, evaluating the impact of the Ugandan Palliative Care Nurse Leadership Programme [22] 18 months following completion, engaging in training oncology nurses and the effectiveness and importance of clinical placements.

### Human rights and engagement on the implementation of the recommendations made by the Uganda Human Rights Commission

An ongoing issue for both cancer and palliative care is that access to care should be a human right [23, 24]. In 2017, the Uganda Human Rights Commission (UHRC) monitored and reported on the right to palliative care in the Annual Report to the Speaker of Parliament of Uganda [25]. Chapter two of the 20th Annual Report details UHRC’s assessment of Palliative Care services, the positive developments, gaps in service provision and makes nine recommendations to government Ministries, Authorities and Departments. The report highlights the achievements made but also points out various gaps in the provision of palliative care services such as the lack of a standalone palliative care policy to guide the implementation of palliative care services, the emphasis on institutionalised care as opposed to home care and low funding for services and others. A workshop was held to engage policy makers on the findings and recommendations made by the UHRC and practical steps were identified for the implementation of the recommendations made.

PCAU also reported back on how they have used the national human rights framework to monitor and report on the right to palliative care, reflecting on the lessons learnt. They noted that it is possible to work within the national human rights framework to bring the issue of palliative care to the fore. Advocacy for palliative care is strengthened when key documents such as the reports of the UHRC mention palliative care and we need to reach all key policy makers in order to influence positive change in the services.

D. K. Musinguzi also presented on the importance of patient dignity as a human right throughout the continuum of care, stressing the importance of patient dignity, something that we can all relate to. She demonstrated this by showing a video of Joseph, a palliative care patient who died this year, who advocated for access to palliative care right up until his death. This video showed the importance of the patient and family voice in our advocacy for access to cancer and palliative care services through UHC.

### Provision of cancer and palliative care to ‘hard to reach’ and ‘vulnerable’ groups

In order to provide UHC and ensure that ‘no one is left behind’ care must be available to all who need it. In the past few years, there has been an emphasis on providing palliative care in humanitarian emergencies and crises. While the focus is rightly on saving lives, there is also an ethical imperative to prevent and relieve pain and suffering; therefore, palliative care should be integrated into our response [26, 27]. Various papers, both oral and posters, reported on work being undertaken with South Sudanese refugees living in the settlements in Adjumani, Northern Uganda. Other hard to reach and vulnerable groups that were discussed included those with psychiatric conditions and those with disabilities such as the deaf. A. Murangira presented a plenary session on access to essential health services for special populations. While representing the deaf, and presenting in sign language, he did not restrict the need for access of cancer and palliative care to the deaf, but to all those with disabilities, ensuring equal access to care and information, through appropriately trained individuals. PCAU has been working for many years with the deaf community in Uganda, with meetings and conferences, such as the PCAU/UCI conference being signed [11].

### Paediatric care

Children requiring cancer and palliative care and their families have unique needs, and it is important that there are services available to them. Thus, paediatric care was a theme that was integrated throughout the conference. Caring for children with cancer and those needing palliative care can be stressful and strategies used by paediatric oncology nurses to cope with the stress of the job at UCI were discussed, along with the lived experience of these nurses for caring for terminally ill children. The nurses lived experiences covered four main themes: attachment, repressed feelings, feeling of helplessness and the need to be supported. The psychosocial difficulties experienced by mothers caring for children were highlighted as were those of children caring for their sick parents. The importance of providing palliative care for children was raised, and Kawempe Home Care shared how the use of their hostels for children and their families requiring cancer care has helped increase access to such care. A study was shared that explored children’s understanding of illness, death and dying, and how this impacts on the care that we provide. UCI also shared the results of some of the cancer care that they are providing for children such as those with Burkitt’s lymphoma, and also discussed how they have found that acute leukaemia and not Burkitt’s lymphoma is the commonest cancer diagnosed at a south-west Uganda paediatric cancer unit.

### Health promotion and prevention

Health promotion and prevention are core components of any cancer control programme [28]. Thus, there was a track addressing these issues throughout the conference. Areas covered included that of screening for cervical cancer, reporting on a systematic review looking at enabling factors for cervical cancer screening as well as a paper on how best to reach rural women with cervical cancer screening and one on the lived experience of patients with advanced cancer of the cervix which identified six themes: socio-demographic profiles of the women; road to cancer diagnosis; effects of disease on women’s quality of life; experiences with mainstream medicine; adapting to having cancer and the needs of the women.

### Policy and advocacy

Having appropriate policies in place is essential for the ongoing development of both cancer and palliative care [29]. The Ministry of Health shared an analysis they had undertaken on the legislative and regulatory threats facing improved access to controlled medicines for patients with life-limiting illnesses in Uganda. This analysis had been undertaken as a direct result of the first cancer and palliative care conference held in 2017 [11], where key stakeholders were engaged at a side-event during the conference when a national committee was formally appointed by the Ministry of Health that undertook this analysis and submitted a detailed report to the Ministry of Health in March 2019 [30]. While the outcome of this report will hopefully be increased access to controlled medicines, it also demonstrated the value of the conference as a platform for initiating ideas to solve barriers to access to cancer and palliative care services.

### Digital technology

Finally, the importance of digital technology in the provision of cancer and palliative care services was recognised and a workshop held. Over the past 5–10 years, the importance of mhealth and the use of digital technology have been recognised globally with numerous projects implemented in this field [31]. PCAU in partnership with the Center for Hospice Care/Hospice Foundation (CHC/HF) and the University of Notre Dame started a mHealth surveillance project in May 2015 to mitigate the issues of lack of accurate and updated information on palliative care services in Uganda. This aimed to obtain and avail credible data to aid evidenced decision-making and thereby enhancing the availability and use of palliative care services in Uganda. The project is now collecting data in 20 facilities across Uganda. Key to scalability and sustainability is the collaboration with the Ministry of Health (MoH) and the integration of this data collection into MoH digital health infrastructure. Digital technology was also utilised throughout the conference in the form of social media, with participants tweeting, putting information on Facebook and Instagram.

## Conclusion

The past few years have seen significant developments in both cancer and palliative care in Uganda, and this was evident in the presentations, and the way that provision has changed and improved since the previous cancer and palliative care conference in 2017 [11]. Emphasis on UHC, along with the support of government and other stakeholders, is important in the ongoing development of cancer and palliative care services in Uganda. Importantly, it was clear throughout that emphasis is not just on coverage, but also on care—what is the quality of the services and care being provided? The success of this second cancer and palliative care conference was evident, and the celebration of all that has been achieved by PCAU in the past 20 years. Collaboration between all organisations is important as we strive to achieve UHC and both PCAU and the UCI need to continue to work alongside the Ministry of Health in order to provide leadership and make sure that we step up and enable UHC for cancer and palliative care to become a reality. As Uganda continues to move forward in the implementation of UHC, and a national health insurance scheme, it is important that we ensure that cancer and palliative care become accessible to all, regardless of age, diagnosis or where you live.

## Conflicts of interest

The authors declare that they have no conflicts of interest.

## Funding

No specific funding was received for the publication of this article.
